# Radiomics‐Based Unsupervised Clustering Identifies Subtypes Associated With Prognosis and Immune Microenvironment in Clear Cell Renal Cell Carcinoma: A Multicenter Study

**DOI:** 10.1002/advs.202506165

**Published:** 2025-06-20

**Authors:** Yusheng Guo, Bingxin Gong, Yi Li, Jie Lou, Shuai Shan, Xiaodong Zhang, Qiang Lu, Dongyong Zhu, Qing Sun, Lianwei Miao, Yuanxi Li, Yu‐Dong Zhang, Wei Tan, Lian Yang, Chuansheng Zheng

**Affiliations:** ^1^ Department of Radiology Union Hospital, Tongji Medical College Huazhong University of Science and Technology Wuhan 430022 China; ^2^ Hubei Provincial Clinical Research Center for Precision Radiology & Interventional Medicine Wuhan 430022 China; ^3^ Hubei Key Laboratory of Molecular Imaging Wuhan 430022 China; ^4^ Department of Radiology the First Affiliated Hospital of Nanjing Medical University Nanjing Jiangsu Province 210009 China; ^5^ Postgraduate Department Shandong First Medical University (Shandong Academy of Medical Sciences) Jinan China; ^6^ Department of Urology the First Affiliated Hospital of Nanjing Medical University Nanjing Jiangsu Province 210009 China; ^7^ Department of Medical Imaging Geriatric Hospital Affiliated with Wuhan University of Science and Technology Wuhan 430065 China

**Keywords:** clear cell renal cell carcinoma, immune microenvironment, prognosis, tumor recurrence, unsupervised clustering

## Abstract

Clear cell renal cell carcinoma (ccRCC) exhibits marked clinical heterogeneity, limiting the prognostic accuracy of traditional staging. We developed an unsupervised radiomics‐based subtyping system integrating multi‐omics data to decode tumor biology and improve risk stratification. Analyzing five cohorts (n = 1700, including surgical cohorts and an advanced ccRCC cohort receiving combined tyrosine kinase inhibitor and immunotherapy [T‐I] treatment), we extracted 1834 CT radiomic features, applying consensus clustering to a discovery cohort (n = 748) and validating across centers. Two subtypes emerged with distinct recurrence risks: Cluster 1 and Cluster 2 (adjusted HR = 2.75 for recurrence, 95% CI 1.42–5.33, P = 0.003). Cluster 2’s high recurrence risk was validated in three external cohorts (adjusted HRs: 1.76, 4.33, and 3.09; all P < 0.05). Radiogenomic analysis revealed Cluster 2 showed a higher frequency of VHL mutations and KDM5C mutations compared to Cluster 1, a more immunosuppressive microenvironment (reduced CD8+ T cell infiltration, P < 0.01; suppressed interferon signaling pathways, Gene Set Enrichment Analysis P < 0.05), and lower PD‐L1 expression. In the T‐I treated advanced ccRCC cohort, Cluster 2 patients had shorter overall survival. This first unsupervised radiomic system stratifies ccRCC by recurrence risk, molecular drivers, and treatment efficacy, offering a framework for precision oncology.

## Graphical abstract

This study developed an unsupervised radiomics system integrating CT imaging and multi‐omics data to stratify clear cell renal cell carcinoma into two subtypes with distinct clinical outcomes. Cluster 1 showed lower recurrence risk and active immunity, while Cluster 2 exhibited higher recurrence risk, enriched VHL/KDM5C mutations, immunosuppressive microenvironment, and poorer response to TKI‐immunotherapy. Validated across cohorts, this approach enhances precision risk stratification and treatment guidance.

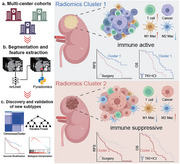

## 1 Introduction

Clear cell renal cell carcinoma (ccRCC) represents 65%–75% of renal malignancies.^[^ ^1^, ^2^ ^]^ Despite advancements in surgical techniques and targeted therapies that have improved outcomes for some patients, the profound molecular heterogeneity of ccRCC continues to drive significant variability in survival outcomes.^[^ ^3^, ^4^ ^]^ Traditional prognostic frameworks, such as TNM staging and WHO/ISUP grading, are limited in their ability to capture the spatial and molecular complexity of tumors.^[^ ^5^, ^6^, ^7^ ^]^ For instance, TNM staging relies on macroscopic tumor characteristics (such size and lymph node involvement) but overlooks microscopic features like angiogenesis, immune infiltration, and metabolic activity, which may be drivers of tumor behavior. Radiomics, a high‐throughput approach to extract quantitative features from medical imaging, has emerged as a promising tool for non‐invasively decoding tumorheterogeneity.^[^ ^8^, ^9^ ^]^ Studies demonstrate that Computed Tomography (CT)‐ or Magnetic resonance imaging (MRI)‐based radiomic features can predict molecular subtypes, metastatic potential, and survival in ccRCC.^[^ ^10^, ^11^, ^12^ ^]^ However, most existing models rely on supervised learning, which restricts classification to predefined clinical labels and risks overlooking latent biological heterogeneity.^[^ ^13^ ^]^ Thus, identifying radiomic subtypes linked to prognosis and tumor microenvironment via unsupervised methods remains a critical unmet need.

Two major challenges hinder progress in ccRCC radiomics research. First, existing models predominantly derive from single‐center studies lacking multi‐institutional validation, thereby compromising clinical generalizability.^[^ ^14^, ^15^ ^]^ Second, mechanistic links between radiomic features and tumor pathobiology has not been fully clarified. For example, abnormal angiogenesis or immune microenvironment characteristics may be indirectly reflected through radiomic patterns, but such connections have not been systematically verified.^[^ ^16^, ^17^ ^]^ Additionally, while combined tyrosine kinase inhibitors (TKIs) and immune checkpoint inhibitors (ICIs) (T‐I therapy) have revolutionized advanced RCC management,^[^ ^18^, ^19^ ^]^ but strategies to non‐invasively identify potential responders remain undefined. Current biomarkers (such as programmed death ligand 1 [PD‐L1] expression, tumor mutation burden [TMB], microsatellite instability [MSI]) rely on invasive tissue sampling and are confounded by spatiotemporal tumor heterogeneity.^[^ ^20^, ^21^ ^]^ A preoperative imaging‐based subtyping system that integrates prognostic prediction with biological insights could optimize holistic ccRCC management.

This multicenter study aims to establishe radiomic subtypes associated with prognosis and immune microenvironment in ccRCC using unsupervised clustering and validate their clinical utility. We analyzed five independent cohorts comprising 1700 ccRCC patients from three medical centers and TCGA, including surgical cohorts and advanced ccRCC cohort receiving T‐I therapy. Consensus clustering uncovered latent radiomic subtypes, validated across centers via a random forest model. Multi‐omics data (gene mutations, immune infiltration scores) and clinical outcomes were integrated to delineate molecular characteristics and therapeutic responses. By bridging radiomic patterns with biological pathways, this study provides generalizable imaging biomarkers and novel insights into tumor heterogeneity, paving the way for personalized surveillance and treatment strategies.

## 2 Experimental Section

### 2.1 Patients

**Figure 1:**
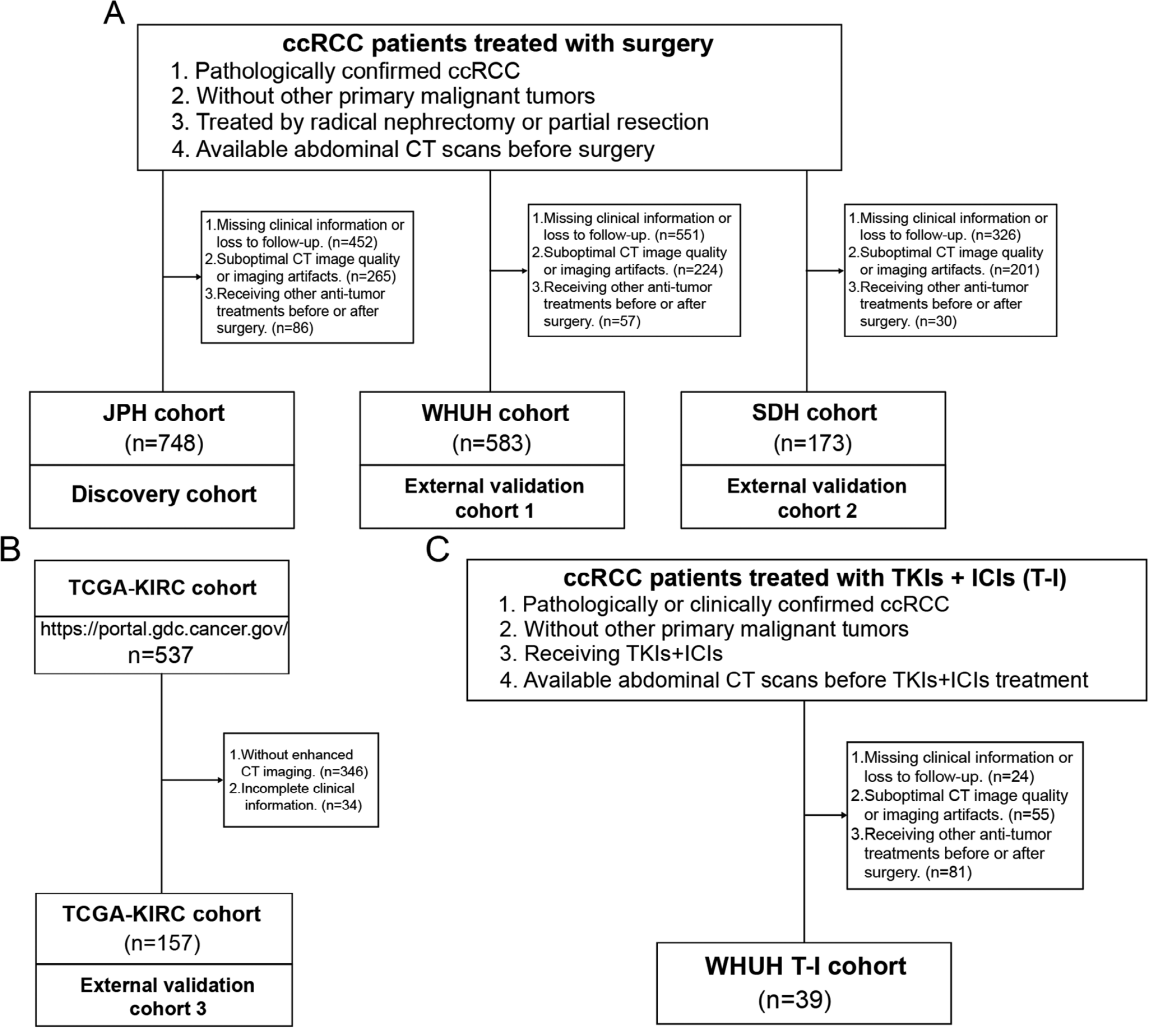
Flowcharts. A) Flowcharts of patient inclusion and exclusion criteria for JPH cohort, WHUH cohort, and SDH cohort. B) Flowcharts of patient inclusion and exclusion criteria for TCGA‐KIRC cohort. C) Flowcharts of patient inclusion and exclusion criteria for WHUH T‐I cohort.

This study was approved by the ethics committees of Union Hospital, Tongji Medical College, Huazhong University of Science and Technology (WHUH), Jiangsu Provincial People's Hospital (JPH), and First Affiliated Hospital of Shandong First Medical University (SDH). Due to its retrospective nature, written informed consent for retrospective datasets was waived. The study adheres to the Transparent Reporting of a multivariable prediction model for Individual Prognosis or Diagnosis (TRIPOD) guideline^[^ ^22^ ^]^ and complies with the Declaration of Helsinki. The inclusion criteria for surgical ccRCC patients were as follows: (1) pathologically confirmed ccRCC; (2) without other primary malignant tumors; (3) treated by radical nephrectomy or partial resection; (4) availability of preoperative abdominal CT scans. Exclusion criteria were: (1) incomplete clinical information or were lost to follow‐up; (2) suboptimal CT image quality or imaging artifacts; (3) receiving other anti‐tumor treatments before or after surgery. We also included a publicly available database (The Cancer Genome Atlas Program [TCGA], https://www.cancer.gov/tcga, TCGA‐KIRC), excluding patients without enhanced CT imaging or with incomplete clinical information, yielding 157 ccRCC patients as an external validation cohort. Additionally, we analyzed a cohort of 39 patients with advanced ccRCC receiving TKIs combined with ICIs. In total, 1700 ccRCC patients across five independent cohorts were included. Detailed inclusion/exclusion criteria and patient recruitment processes for each cohort are shown in **Figure** **1**.

### 2.2 Pathological Evaluation

All resected samples underwent routine clinical histopathological examination and analysis, including hematoxylin & eosin staining for histological classification. For included ccRCC pathology slides, WHO/International Society of Urological Pathology (ISUP) grading was performed by cell nuclear characteristics.

### 2.3 Follow‐up Protocol and Clinical Outcome Assessment

Postoperative patients were followed via telephone calls or outpatient imaging evaluations. Follow‐up was conducted every two months within the first six months post‐surgery, then every three to four months. For patients receiving T‐I therapy, imaging examinations were performed 4–8 weeks after initial treatment. Short‐term efficacy was evaluated using RECIST 1.1,^[^ ^23^ ^]^ categorized as partial response (PR), complete response (CR), stable disease (SD), or progressive disease (PD), with objective response rate (ORR) and disease control rate (DCR) determined. Follow‐up imaging was subsequently performed every 1–2 months. Recurrence‐free survival (RFS) was defined as the time from surgery to tumor recurrence. Progression‐free survival (PFS) calculated from the start of systemic therapy to disease progression or death from any cause. Overall survival (OS) was defined as the time from treatment start to death from any cause.

### 2.4 CT Scan Parameters and Radiomic Feature Extraction

Contrast‐enhanced abdominal enhanced CT scans were performed using a unified protocol across participating centers to ensure consistency. Detailed scanner information are provided in Table S1 (Supporting Information). All CT images were retrieved from the Picture Archiving and Communication System, with images and parameters from the (The Cancer Imaging Archive) TCIA website (https://www.cancerimagingarchive.net/collection/tcga‐kirc/) also utilized. Tumor segmentation was performed using a pre‐trained nnU‐Net‐based model, optimized for RCC, generating 3D volumetric masks from arterial‐phase images.^[^ ^24^ ^]^ Two experienced intermediate radiologists, Q.S. (17 years of abdominal disease diagnosis experience) and D.Y.Z. (10 years of experience), verified and adjusted the tumor masks. Q.S. handled the JPH and WHUH cohorts, while D.Y.Z. managed the TCGA‐KIRC and WHUH T‐I cohorts. To mitigate technical variability across imaging platforms, all volumetric datasets underwent standardized preprocessing. First, volumetric resampling was performed using trilinear interpolation to achieve isotropic 1×1×1 mm^3^ voxel spacing. Additionally, a lung window normalization protocol (window width: 300 HU; window level: 50 HU) was applied to the voxel intensity values to enhance lesion contrast. The “Pyradiomics” toolkit was used to extract 1834 radiomic features from arterial‐phase tumors, categorized into five groups: (1) first‐order features, (2) shape features, (3) texture features, (4) wavelet features, and (5) Laplacian of Gaussian (LoG) features. For the WHUH T‐I cohort, only the primary tumor was segmented for radiomic feature extraction.

### 2.5 Unsupervised Clustering Analysis

For the 1834 radiomic features, data were scaled using the “scale” function in R, and batch effects were corrected with the “ComBat” harmonization algorithm, principal component analysis (PCA) demonstrates that the radiomics features exhibit robustness across different cohorts (Figure S1, Supporting Information). For the JPH cohort, consensus clustering was performed using the “ConsensusClusterPlus” R package with the K‐means method (Euclidean distance), repeated 1500 times to ensure classification stability (pItem = 0.8 and pFeature = 1). The optimal number of clusters was determined by the relative change in the area under the cumulative distribution function curve, and PCA was used to validate the distinct radiomic cluster components. A random forest (RF) classifier was then trained on 70% of JPH cohort patients (training cohort) using all 1834 radiomic features (ntree = 500, splitting criteria = “gini”), with the remaining 30% were used as the testing cohort. Accuracy, sensitivity, and specificity are evaluation metrics for model performance. This trained model was subsequently applied to assign cluster labels to samples in other cohorts.

### 2.6 Biological Background Analysis of Radiomic Subtypes

Somatic mutation landscapes in TCGA‐KIRC cohort were analyzed using maftools, while TMB and MSI scores were obtained using the “MANTIS” and “maftools” packages. Immune infiltration was calculated based on RNA‐seq data using CIBERSORT. Genes associated with radiomic clusters were identified via Spearman rank correlation test, with Benjamini‐Hochberg correction for multiple testing. Gene Set Enrichment Analysis (GSEA) was performed for interferon‐related pathways (“Interferon Signaling”, “Interferon Gamma Signaling”, and “Interferon Alpha Beta Signaling”) and antigen presentation‐related pathways (“Antigen Processing Cross Presentation”, “Antigen Presentation Folding Assembly and Peptide Loading of Class I Mhc”, and “Class I Mhc Mediated Antigen Processing Presentation”).

### 2.7 Statistical Analysis

Continuous variables were presented as mean ± standard deviation or median (interquartile range [IQR]), and categorical variables as frequency and percentage. Comparisons between continuous variables were conducted using Wilcoxon rank‐sum tests or Student's t‐tests, and categorical variables were analyzed with chi‐square tests or Fisher's exact tests as appropriate. Kaplan‐Meier survival curves and Log‐rank test were used to compare survival differences between groups. Proportional hazard assumptions were validated to select suitable variables for Cox regression modeling. Multivariate analyses included variables with low multicollinearity (variance inflation factor < 4), and hazard ratios (HRs) with 95% confidence intervals (CIs) were calculated to assess independent prognostic significance for recurrence‐free survival (RFS) or overall survival (OS). Statistical analysis and graphing were performed using R (version 4.1.0), SPSS (version 26.0), and Graph Prism 8, with p‐values < 0.05 (two‐sided) considered statistically significant.

### 2.8 Ethics Approval and Consent to Participate

The ethical approvals for this study were provided by the Institutional Review Board of Union Hospital, Tongji Medical College, Huazhong University of Science and Technology, the Institutional Review Board of Jiangsu Provincial People's Hospital and the Institutional Review Board of First Affiliated Hospital of Shandong First Medical University.

## 3 Results

### 3.1 Baseline Characteristics of Patients

This study included 1700 ccRCC patients from 3 medical centers and a public database. Table S1 (Supporting Information) outlines the clinical characteristics of the discovery cohort (JPH cohort, n = 748), external validation cohort 1 (WHUH cohort, n = 583), external validation cohort 2 (SDH cohort, n = 173), and external validation cohort 3 (TCGA‐KIRC, n = 157). Among these patients, 567 were female (34.13%), and 1274 (76.70%) had a WHO/ISUP grade of 1–2. Table S2 (Supporting Information) presents the characteristics of the cohort receiving TKIs and ICIs (WHUH T‐I cohort, n = 39).

### 3.2 Two Radiomic Subtypes of ccRCC

**Figure 2:**
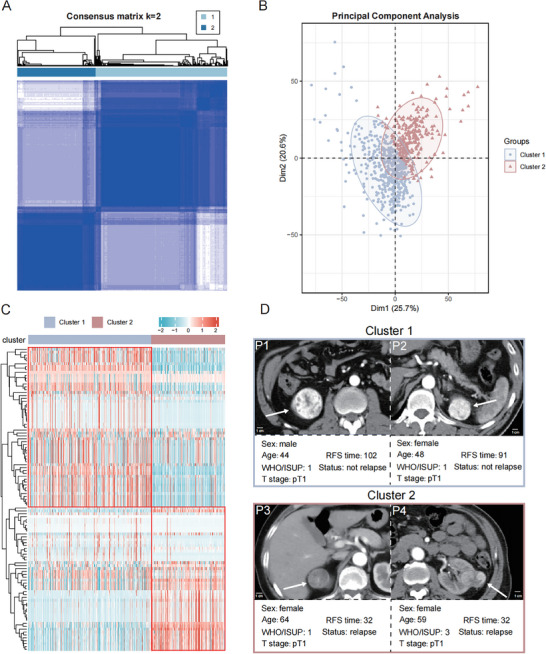
Two Radiomic Subtypes of ccRCC. A) Consensus matrix of 748 ccRCC patients in discovery cohort when k  =  2 using K‐means clustering (Euclidean distance and 1500 iterations). B) PCA visualization confirms distinct separation between Cluster 1 (red) and Cluster 2 (blue). C) The heatmap of Z‐score‐normalized original radiomic features (n = 105) in JPH cohort between two radiomic subtypes (Cluster 1 and Cluster 2). Each column represents a patient, and each row represents a specific radiomic feature. D) Representative CT imaging with clinical information from different clusters.

Unsupervised consensus clustering of 1834 radiomic features robustly stratified the discovery cohort into two subtypes when K = 2 based on the decline in cumulative distribution function area under the curve (**Figure** **2**A; Figure S2, Supporting Information). PCA revealed significant differences between these clusters (Figure 2B). Further re‐clustering of 105 original features highlighted distinct patterns (Figure 2C): Cluster 1 is now described as relatively enhanced and relatively homogeneous, while Cluster 2 is described as relatively weakly enhanced and relatively heterogeneous (Figure 2D). After reviewing the pathological data, we found that the proportion of tumors with hemorrhage, necrosis, and cystic changes was higher in Cluster 2 patients (Figure S3, Supporting Information).

### 3.3 Radiomic Subtypes as Independent Predictors of Recurrence

**Figure 3:**
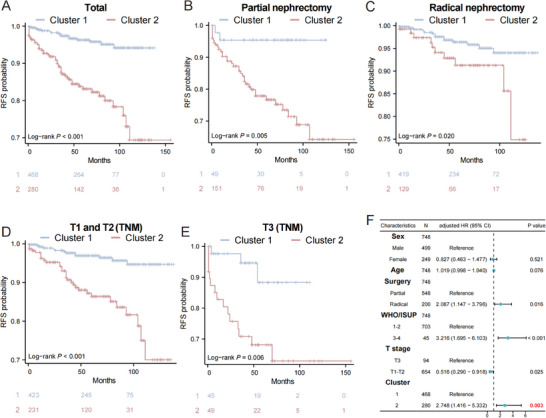
Radiomic Subtypes as Independent Predictors of Recurrence. A) The Kaplan‐Meier survival curves compare RFS between Cluster 1 (n = 468) and Cluster 2 (n = 280) in the total JPH cohort (unadjusted HR = 4.98, 95% CI:2.95‐8.39, P < 0.001). B) The Kaplan‐Meier survival curves compare RFS between Cluster 1 (n = 49) and Cluster 2 (n = 151) in patients treated with partial nephrectomy (unadjusted HR = 5.95, 95% CI:1.43‐25, P = 0.005). C) The Kaplan‐Meier survival curves compare RFS between Cluster 1 (n = 419) and Cluster 2 (n = 129) in patients treated with radical nephrectomy (unadjusted HR = 2.54, 95% CI:1.13‐5.73, P = 0.020). D) The Kaplan‐Meier survival curves compare RFS between Cluster 1 (n = 423) and Cluster 2 (n = 231) in T1‐2 ccRCC patients (unadjusted HR = 4.55, 95% CI:2.37‐8.73, P < 0.001). E) The Kaplan‐Meier survival curves compare RFS between Cluster 1 (n = 45) and Cluster 2 (n = 49) in T3 ccRCC patients (unadjusted HR = 4.83, 95% CI:1.40‐16.72, P = 0.0129). F) Forest plot summarized independent predictors of RFS after adjusting for clinical information and Radiomic Cluster emerged a strong prognostic factor.

Kaplan‐Meier survival curves with log‐rank tests showed statistically significant differences in recurrence times between Cluster 1 and Cluster 2 (P < 0.001, **Figure** **3**A). This difference was consistent across patients undergoing partial nephrectomy (P = 0.005, Figure 3B) or radical nephrectomy (P = 0.020, Figure 3C), as well as between T1‐T2 and T3 tumor stages (P = 0.006 and P < 0.001, Figure 3D,E). Multivariate Cox proportional hazards modeling further established radiomic clusters as independent prognostic factors for recurrence (adjusted HR: 2.75, 95% CI [1.42‐5.33], P = 0.003, Figure 3F).

### 3.4 External Validation of Radiomic Subtypes

**Figure 4:**
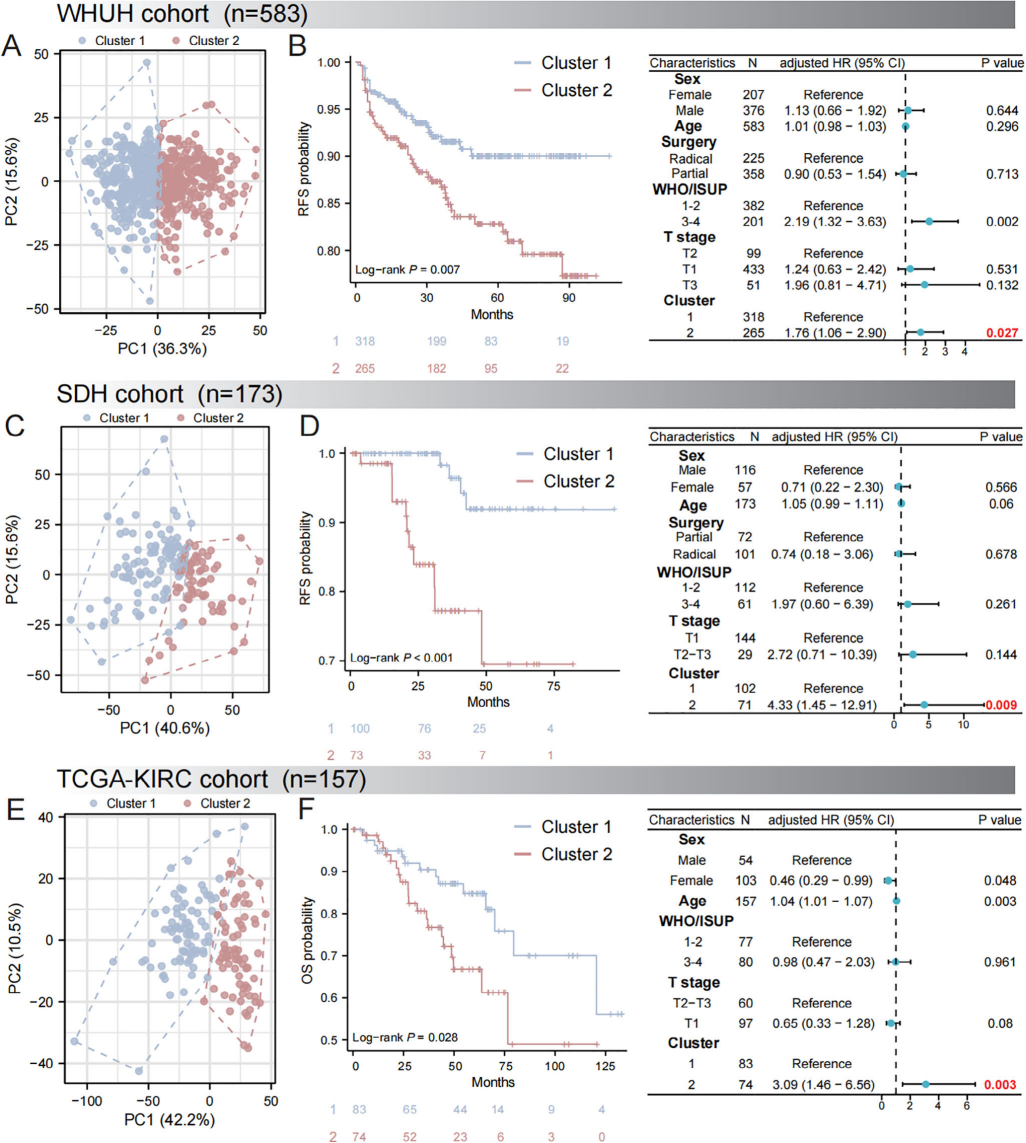
External Validation of Radiomic Subtypes. A,C,E) Principal Component Analysis validation of radiomic clusters. B,D,F) Prognostic validation via survival and multivariate Cox analysis. In the WHUH cohort, Kaplan‐Meier curves demonstrated significantly shorter RFS in Cluster 2 (unadjusted HR = 1.95, 95% CI:1.19‐3.19, P = 0.007). Multivariate Cox regression adjusted for clinical information confirmed Cluster 2 as an independent risk factor for RFS (adjusted HR = 1.76, 95% CI: 1.06‐2.90, P = 0.027). In the SDH cohort, Kaplan‐Meier curves demonstrated significantly shorter RFS in Cluster 2 (unadjusted HR = 3.57, 95% CI:1.26‐10.13, P<0.001). Multivariate Cox regression adjusted for clinical information confirmed Cluster 2 as an independent risk factor for RFS (adjusted HR = 4.33, 95% CI: 1.45‐12.91, P = 0.009). In the TCGA‐KIRC cohort, Kaplan‐Meier curves demonstrated significantly shorter OS in Cluster 2 (unadjusted HR = 2.14, 95% CI:1.07‐4.28, P = 0.028). Multivariate Cox regression adjusted for clinical information confirmed Cluster 2 as an independent risk factor for OS (adjusted HR = 3.09, 95% CI: 1.46‐6.56, P = 0.003).

A RF model trained on the discovery cohort (70% training, n = 524; 30% testing, n = 224) demonstrated excellent performance in subtype prediction (100% in training cohort, 96.0% in testing cohort; Figure S4A,B, Supporting Information). From the perspective of feature importance (Figure S5, Supporting Information), we found that radiomic features such as gradient_gldm_DependenceNonUniformityNormalized and gradient_glcm_InverseVariance were the main factors driving the classification. This indicates that intratumoral structural density heterogeneity is closely related to the two radiomic subtypes. In WHUH cohort, we found that PCA validated significant differences between two radiomic clusters (**Figure** **4**A), and cluster 2 had a shorter RFS (Figure 4B), which was an independent predictor of RFS (adjusted HR: 1.76, 95% CI [1.06‐2.90], P = 0.027). Similarly, in SDH cohort, PCA validated significant differences in radiomic clusters (Figure 4C), and cluster was also an independent predictor of RFS (adjusted HR: 4.33, 95% CI [1.45–12.91], P = 0.009, Figure 4D). In TCGA‐KIRC, radiomic subtypes independently predicted overall survival (adjusted HR: 3.09, 95% CI [1.46–6.56], P = 0.003,Figure 4E,F). Overall, the prognostic significance of radiomic subtypes was validated across three external cohorts. In addition, to confirm the stability of the results, we combined three Asian cohorts (n = 1504) and performed clustering using the same parameters (Figure S6A, Supporting Information). When K = 2, the cumulative distribution function area under the curve declined sharply, leading to the identification of new Cluster 1 and Cluster 2 (Figure S6B, Supporting Information). We found that these new subtypes could also significantly stratify the RFS of patients (Figure S6C, Supporting Information), and after adjusting for clinical factors, the new radiomic subtypes remained independent predictors (adjusted HR: 1.59, 95% CI [1.11–2.27], P = 0.011, Figure S6D, Supporting Information).

### 3.5 Biological Background of Radiomic Subtypes

**Figure 5:**
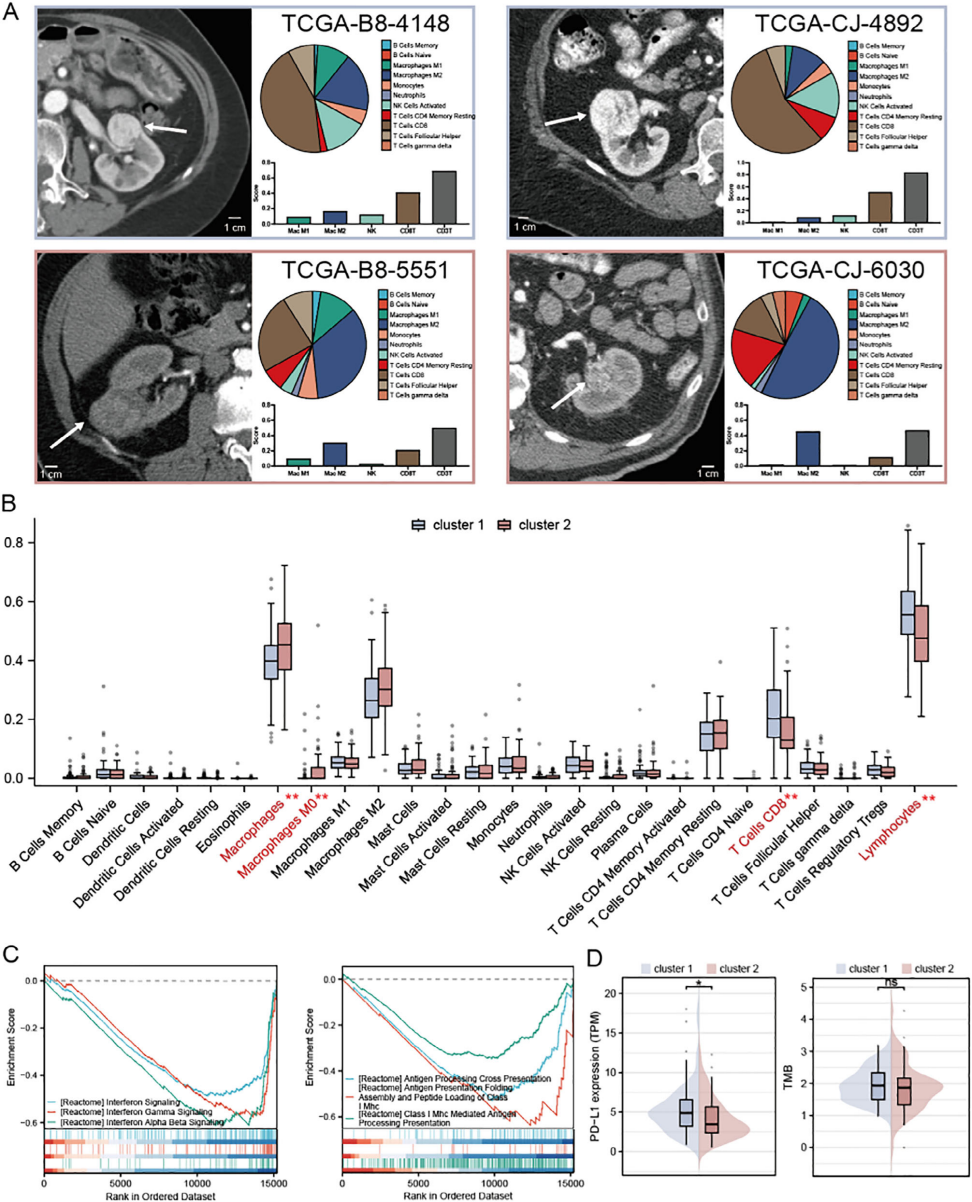
Biological Background of Radiomic Subtypes. A) Four representative patients with CT imaging and immune infiltration information (Cluster 1 were upper panels and Cluster 2 were lower panels). B) The difference of immune infiltration (derived from CIBERSORT analysis) between Cluster 1 and Cluster 2. C) The Gene Set Enrichment Analysis reveals different status of interferon pathway and antigen presentation pathway between Cluster 1 and Cluster 2. D) The difference of PD‐L1 expression and TMB status between Cluster 1 and Cluster 2.

We integrated genomic and transcriptomic data from TCGA to explore the biological mechanisms driving radiomic subtypes. Analysis of the genomic landscape revealed significant differences between clusters: Cluster 2 exhibited higher frequencies of VHL mutations (76.2% vs 50.0%, P = 0.03) and KDM5C mutations (2.0% vs 16.7%, P = 0.04) compared to Cluster 1 (Figure S7, Supporting Information). As previously reported, VHL mutations are associated with hypoxia activation and NK cell inhibition, potentially contributing to poor prognosis.^[^ ^25^, ^26^ ^]^ The KDM5C mutation may be related to chromatin remodeling and associated with the invasiveness of tumor cells and reduced survival of patients.^[^ ^27^ ^]^ Subsequent analysis of immune infiltration patterns showed distinct profiles between clusters. Among four representative cases, Cluster 1 patients demonstrated increased CD3+ T cells, CD8+ T cells, NK cells, and M1 macrophages, whereas Cluster 2 patients displayed inverse trends (**Figure** **5**A). Quantitative analysis confirmed significantly elevated macrophage (P<0.01) and macrophage M0 (P < 0.01) infiltration in Cluster 2, while Cluster 1 showed higher levels of CD8+ T cells (P < 0.01) and lymphocytes (P < 0.01) (Figure 5B). GSEA revealed marked suppression of interferon signaling and antigen presentation pathways in Cluster 2 (Figure 5C). Consistent with this, TCR signaling activation status differed significantly between clusters (Figure S8, Supporting Information). In addition, we found that interferon_gamma_response and interferon_alpha_response were significantly activated in Cluster 1 tumors (Figure S9A, Supporting Information), ranking as the top 2 among the top 10 pathways (Figure S9B, Supporting Information). We examined the biomarkers of immune checkpoint inhibitors currently used in clinical practice, including PD‐L1 expression, TMB, and MSI. PD‐L1 expression showed a significant increase in cluster 1, while TMB and MSI were higher in Cluster 1, although not statistically significant (Figure 5D; Figure S10, Supporting Information). These findings suggest distinct biological and immunological profiles underlying the radiomic subtypes, with potential implications for therapeutic response.

### 3.6 Association of Radiomic Subtypes with Prognosis in Advanced ccRCC Patients Receiving T‐I

**Figure 6:**
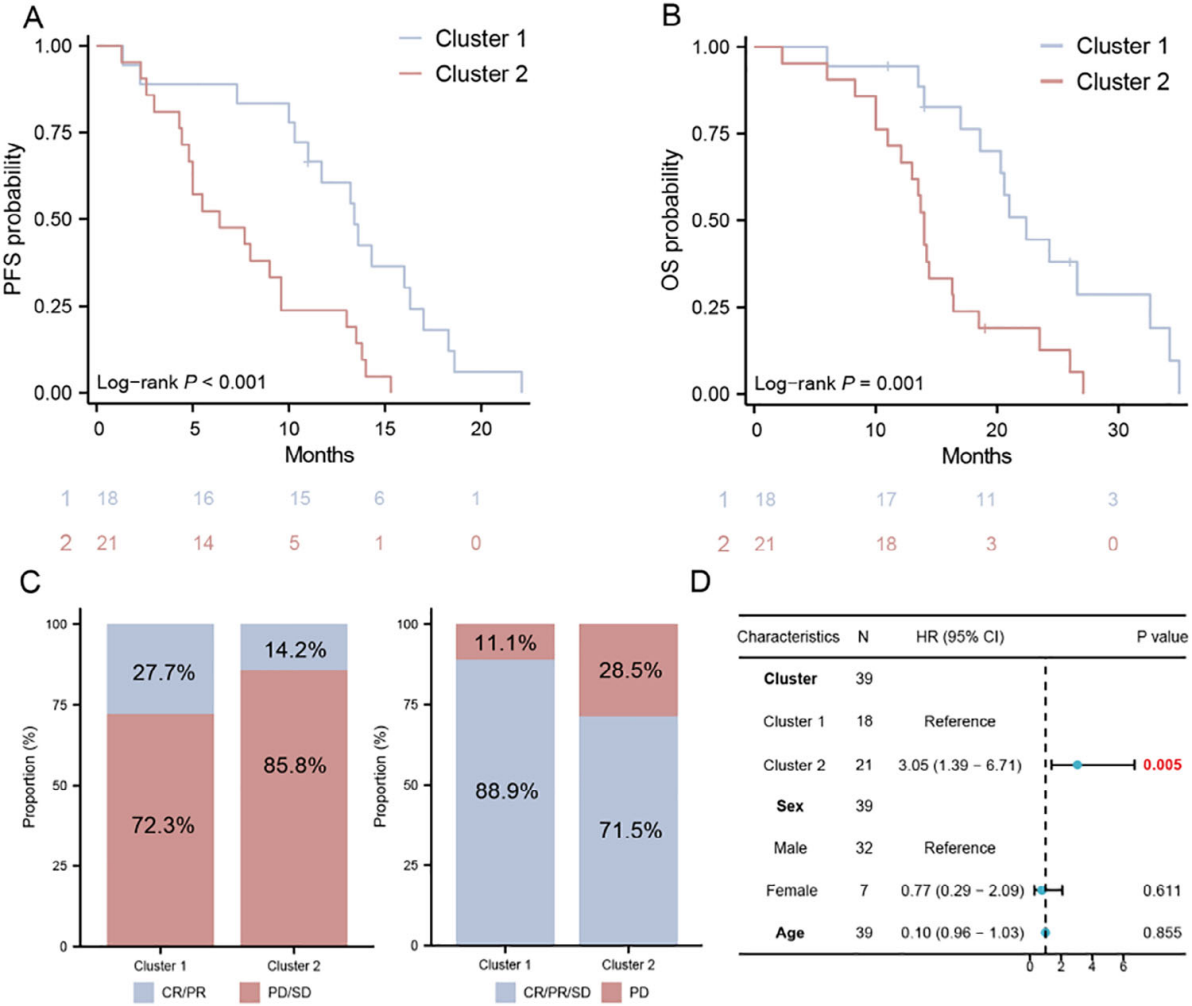
Association of Radiomic Subtypes with Prognosis in Advanced ccRCC Patients Receiving T‐I. A) Kaplan‐Meier curves demonstrated significantly shorter PFS in Cluster 2 (unadjusted HR = 3.80, 95% CI:1.74‐8.26, P<0.001). B) Kaplan‐Meier curves demonstrated significantly shorter OS in Cluster 2 (unadjusted HR = 3.25, 95% CI:1.52‐6.90, P = 0.001). C) The ORR and DCR between Cluster 1 and Cluster 2. D) Multivariate Cox regression adjusted for clinical information confirmed Cluster 2 as an independent risk factor for OS (adjusted HR = 3.05, 95% CI: 1.39‐6.71, P = 0.005).

In the current Chinese clinical guidelines, the combination of TKIs and ICIs (T‐I) is considered a promising treatment strategy for advanced RCC. Based on the aforementioned findings, which revealed that Cluster 1 is associated with immune infiltration and PD‐L1 expression, and considering that VHL mutations may be linked to hypoxia activation and angiogenesis, it is meaningful to analyze the association between radiomic subtypes and outcomes in patients with unresectable RCC treated with TKIs and ICIs. In the WHUH T‐I cohort of 39 patients receiving TKIs and ICIs, we found that Cluster 2 was significantly associated with shorter PFS (P < 0.001) and OS (P = 0.001, **Figure** **6**A,B). Although Cluster 2 was also associated with lower ORR and DCR, these differences were not statistically significant (Figure 6C). After adjusting for age and sex, radiomic subtypes remained an independent predictor of OS (adjusted HR: 3.05, 95% CI [1.39–6.71], P = 0.005, Figure 6D).

## 4 Discussion

This multicenter study utilizing unsupervised clustering analysis has, for the first time, established a radiomic subtype classification system demonstrating significant associations with prognosis and immune microenvironment features ccRCC. Cross‐cohort validation based on 1700 patients demonstrated that Cluster 2 exhibited increased recurrence risk in surgical cohorts (HR: 2.75, 95% CI [1.42–5.33], P = 0.003) and was associated with shorter PFS and OS in advanced ccRCC patients receiving T‐I therapy. Compared to conventional TNM staging and histopathological grading systems, radiomic subtypes offer a non‐invasive imaging approach capable of comprehensively capturing tumor heterogeneity through 3D volumetric analysis, thereby providing a more holistic representation of tumor biology. Notably, after adjusting for T stage or WHO/ISUP grade in each cohort, radiomic subtypes remained significantly associated with RFS, suggesting its potential to complement (rather than replace) existing frameworks through orthogonal biological insights.

Previous studies have explored the associations between RCC imaging features and clinical outcomes. Udayakumar et al. reported that ccRCC samples with high MRI enhancement may respond better to anti‐angiogenic therapy, though the sample size was small.^[^ ^16^ ^]^ Xiong et al. developed a deep learning model to differentiate aggressive from indolent renal tumors using preoperative CT images, demonstrating that radiomics‐based models could effectively stratify prognosis.^[^ ^28^ ^]^ Similarly, our analysis identified comparable tumor subtypes with distinct prognostic profiles. Goh et al. further showed that CT texture features could predict treatment response and disease progression in TKI‐treated RCC patients, exhibiting superior predictive accuracy compared to conventional RECIST and Choi criteria.^[^ ^29^ ^]^ Collectively, these findings suggest potential of radiomic features as robust prognostic biomarkers in RCC management.

Our radiogenomic analysis reveals that the identified radiomic subtypes are associated with distinct molecular and immunological mechanisms. Specifically, Cluster 2 demonstrated higher frequencies of VHL and KDM5C mutations. VHL mutation drives angiogenesis and immune suppression through hypoxia‐inducible factor (HIF) pathway activation, while KDM5C mutation promote tumor invasiveness via epigenetic dysregulation. These molecular features align with the poorer prognosis observed in Cluster 2 patients. Immune profiling further highlighted divergent tumor microenvironments between subtypes. Cluster 1 tumors exhibited increased infiltration of CD8+ T cells and natural killer (NK) cells, whereas Cluster 2 was characterized by elevated M0 macrophage populations. This difference suggests that radiomic subtypes may serve as indirect indicators of tumor immunological heterogeneity. GSEA further indicated suppressed interferon signaling and antigen presentation pathways in Cluster 2 patients, potentially impairing immune recognition and clearance of tumors and accelerating disease progression. These findings provide direct evidence linking radiomics to tumor biology and suggest that imaging features may serve as non‐invasive biomarkers for assessing tumor immune microenvironments. Notably, radiomic subtypes also predict treatment response in advanced ccRCC patients. In the T‐I cohort, Cluster 2 patients had significantly shorter PFS and OS. Although differences in objective ORR and DCR were not statistically significant, this trend aligns with the immune‐suppressive microenvironment observed in Cluster 2. For example, higher PD‐L1 expression in Cluster 1 and fewer VHL mutations may be associated with reduced HIF pathway activation, potentially correlating with contrast agent uptake in imaging features. Studies have reported that hypoxic microenvironments are linked to immunosuppressive states,^[^ ^30^, ^31^ ^]^ while tumor vascularity is associated with improved anti‐angiogenic therapy efficacy.^[^ ^32^ ^]^ Consequently, Cluster 1 tumors may represent an optimal candidate population for TKI+ICI combination therapy. Conversely, hypoxia in Cluster 2 tumors, driven by VHL mutations and HIF pathway activation, may impair vascular normalization, likely contributing to reduced CT contrast enhancement. This radiomic classification mirrors established transcriptomic‐based ccRCC molecular subtypes, including subtypes characterized by concurrent immune activation and angiogenesis,^[^ ^33^, ^34^, ^35^ ^]^ as well as hypoxic or immune‐desert subtypes. Studies demonstrate that hypoxia inhibitors can reverse hypoxia‐associated immune exclusion by normalizing tumor vasculature and enhancing CD8+ T cell infiltration.^[^ ^36^, ^37^ ^]^ Integrating HIF inhibitors with ICIs may thus rescue immunotherapy efficacy in Cluster 2 patients. From the perspective of adaptive immune resistance theory,^[^ ^38^ ^]^ the relative homogeneity within the tumor may facilitate T‐cell penetration. Under these conditions, tumors may upregulate PD‐L1 expression in response to immune pressure as an immune escape mechanism,^[^ ^38^ ^]^ Our analysis of TCGA transcriptomic data revealed that Cluster 1 exhibits active interferon signaling, and since interferon‐gamma can induce PD‐L1 expression,^[^ ^39^ ^]^ this may ultimately manifest as distinct imaging features.

This study has several strengths. First, the use of unsupervised clustering to explore radiomic features avoids the limitations of supervised learning, where classification is constrained by predefined clinical labels, enabling a more objective discovery of underlying biological heterogeneity. Second, the integration of multicenter data and TCGA‐KIRC database, encompassing diverse treatment modalities and clinical settings, ensures the reliability and generalizability of the findings. Additionally, this CT‐based approach is non‐invasive and cost‐effective, offering potential benefits for resource‐limited regions.

However, there are some limitations. First, although data from multiple centers were included, the sample size remains relatively limited, particularly in the T‐I cohort, which may affect result stability. Future validation studies should prioritize larger, more diverse cohorts across different treatment paradigms to confirm the generalizability of radiomic subtypes. Second, radiomic feature extraction is based on CT images, which, while widely used clinically, have limited resolution and soft tissue contrast, potentially impacting feature accuracy. Future research could explore combining other imaging modalities (such as MRI and PET‐CT) to extract richer radiomic features and improve model performance. Finally, although associations between radiomic subtypes and immune microenvironment features were observed, direct biological validation remains pending. Techniques such as single‐cell or spatial transcriptomics or multiplex immunofluorescence mapping could provide critical spatial contextualization of immune cell distributions relative to radiomic subtypes, thereby strengthening the mechanistic understanding of imaging‐biology correlations. These investigations will be essential for translating radiomic findings into clinically actionable insights.

## 5 Conclusions

In summary, this study, through unsupervised clustering of radiomic features, has uncovered the heterogeneity of ccRCC and established a subtype classification system closely associated with clinical outcomes and biological characteristics. Clinically, this system enables personalized risk‐adapted management: Cluster 2 patients may benefit from intensified surveillance and novel hypoxia inhibitor combinations, while Cluster 1 patients are optimal candidates for TKI‐ICI therapy.

## Conflict of Interest

The authors declare no conflict of interest.

## Author Contributions

Y.S.G., B.X.G., Y.L., and J.L. contributed equally to this work. Y.S.G., B.X.G., Y.L., and J.L. conceived and designed the study, and were involved in data interpretation. Y.S.G., B.X.G., S.S., X.D.Z., Q.L., D.Y.Z., Q.S., L.W.M., Y.X.L., and Y.D.Z. participated in data acquisition and analysis. Y.S.G. and B.X.G. drafted the initial manuscript. C.S.Z., L.Y., and W.T. jointly supervised the project and critically revised the manuscript for intellectual content.

## Supporting information



Supporting Information

## Data Availability

The data that support the findings of this study are available from the corresponding author upon reasonable request.
