# Supplementary material for: Radiomics‐Based Unsupervised Clustering Identifies Subtypes Associated With Prognosis and Immune Microenvironment in Clear Cell Renal Cell Carcinoma: A Multicenter Study
Source: Adv Sci (Weinh). 2025 Jun 20;12(34):e06165. doi: 10.1002/advs.202506165 (PMC12442619; doi:10.1002/advs.202506165)
Supplement: Supplementary file 1 — Supporting Information [file ADVS-12-e06165-s001.docx]

**Supplementary Material**

**Radiomics-based Unsupervised Clustering Identifies Subtypes Associated with Prognosis and Immune Microenvironment in Clear Cell Renal Cell Carcinoma: A Multicenter Study**

**Table of Contents**

**[Supplementary Material 1](#_Toc25893)**

**[Supplementary Figures and Figure legends 3](#_Toc27944)**

[Figure S1. PCA across different cohorts 3](#_Toc14889)

[Figure S2. Cumulative distribution function 4](#_Toc28238)

[Figure S3. The proportion of tumors with hemorrhage, necrosis, and cystic changes 5](#_Toc11262)

[Figure S4. The training and performance of random forest. 6](#_Toc16634)

[Figure S5. Random forest feature importance 7](#_Toc22416)

[Figure S6. Re-clustering in combined cohort 8](#_Toc5148)

[Figure S7. The top 20 mutated genes were visualized by waterfall plot 9](#_Toc23153)

[Figure S8. GSEA showed downregulation of TCR Signaling pathways in Cluster 2 10](#_Toc18235)

[Figure S9. GSEA of Hallmark gene sets 11](#_Toc18727)

[Figure S10. The difference of MSI MANTIS Score between Cluster 1 and Cluster 2 12](#_Toc18105)

**[Supplementary Tables 13](#_Toc28533)**

[Table S1. CT scanners and parameters of the three cohorts 13](#_Toc20118)

[Table S2. Baseline information of WHUH T-I cohort 15](#_Toc11300)

## Supplementary Figures and Figure legends

## Figure S1. PCA across different cohorts

##
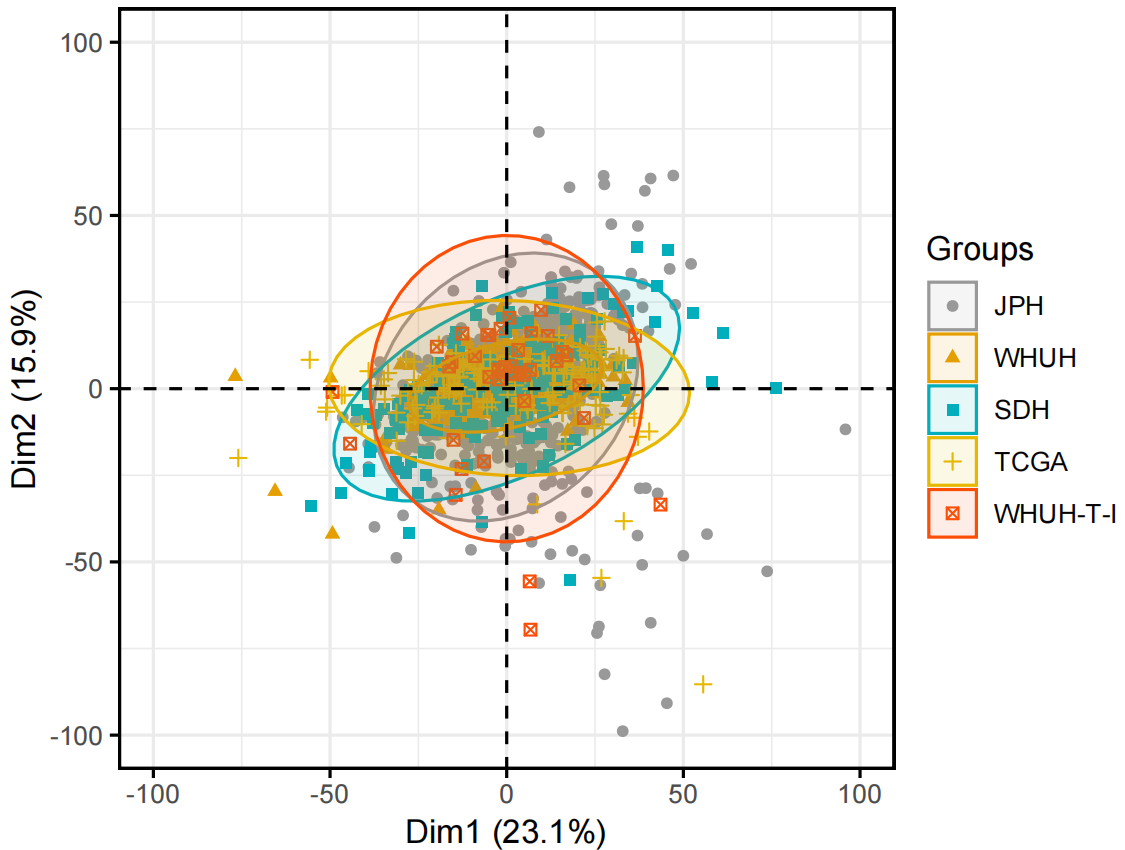


**Figure S1.** PCA demonstrates that the radiomics features exhibit robustness across different cohorts.

## Figure S2. Cumulative distribution function


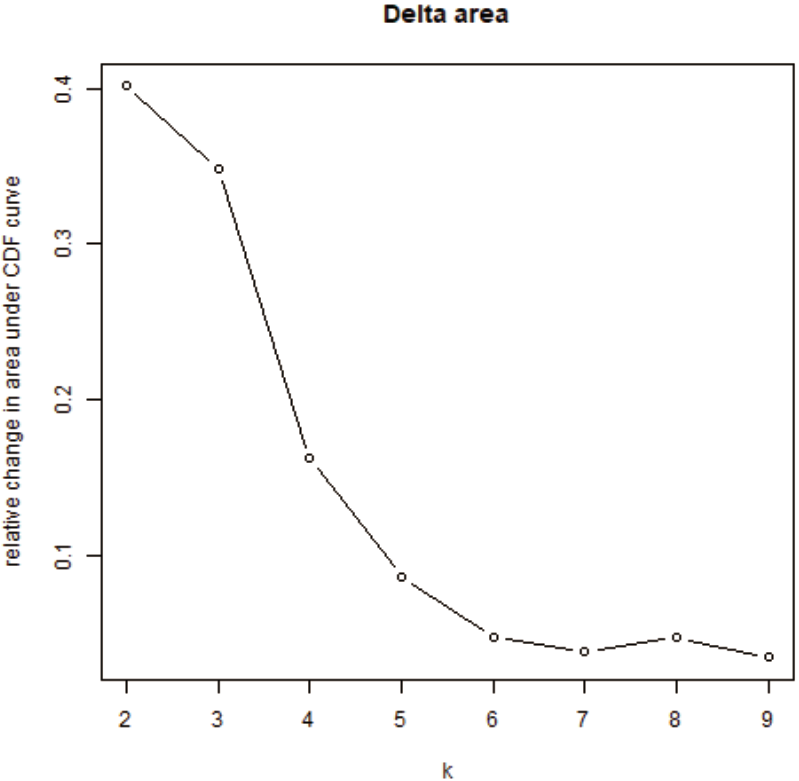


**Figure S2.** Cumulative distribution function.

## Figure S3. The proportion of tumors with hemorrhage, necrosis, and cystic changes


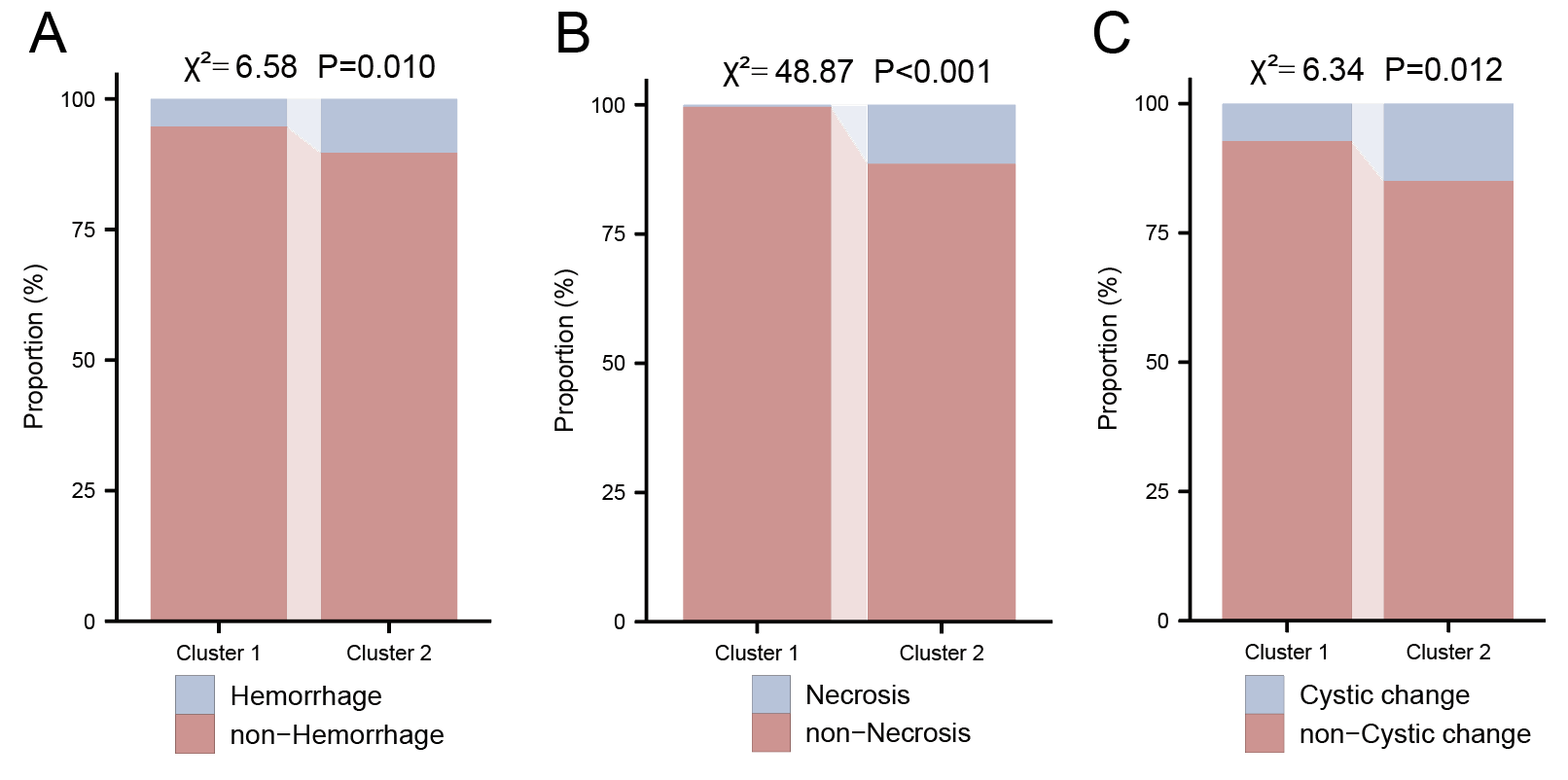


**Figure S3.** The proportion of tumors with hemorrhage (A), necrosis (B), and cystic changes (C) in different Clusters.

## Figure S4. The training and performance of random forest.


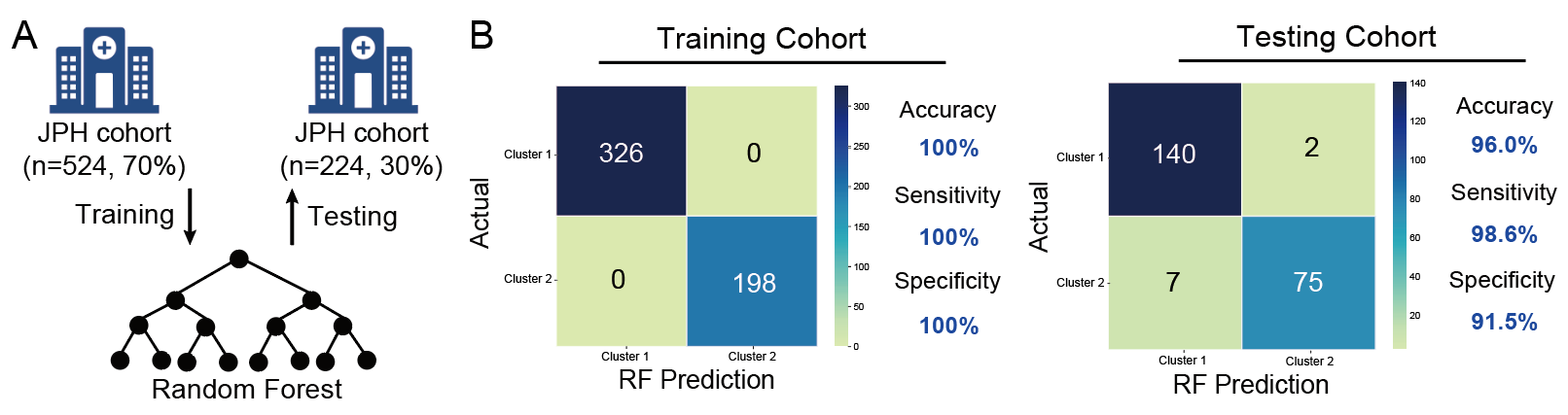


**Figure S4.** (A) The training of random forest to predict Cluster 1 and Cluster 2 in the JPH cohort. (B)The performance of random forest in training cohort and testing cohort.

## Figure S5. Random forest feature importance


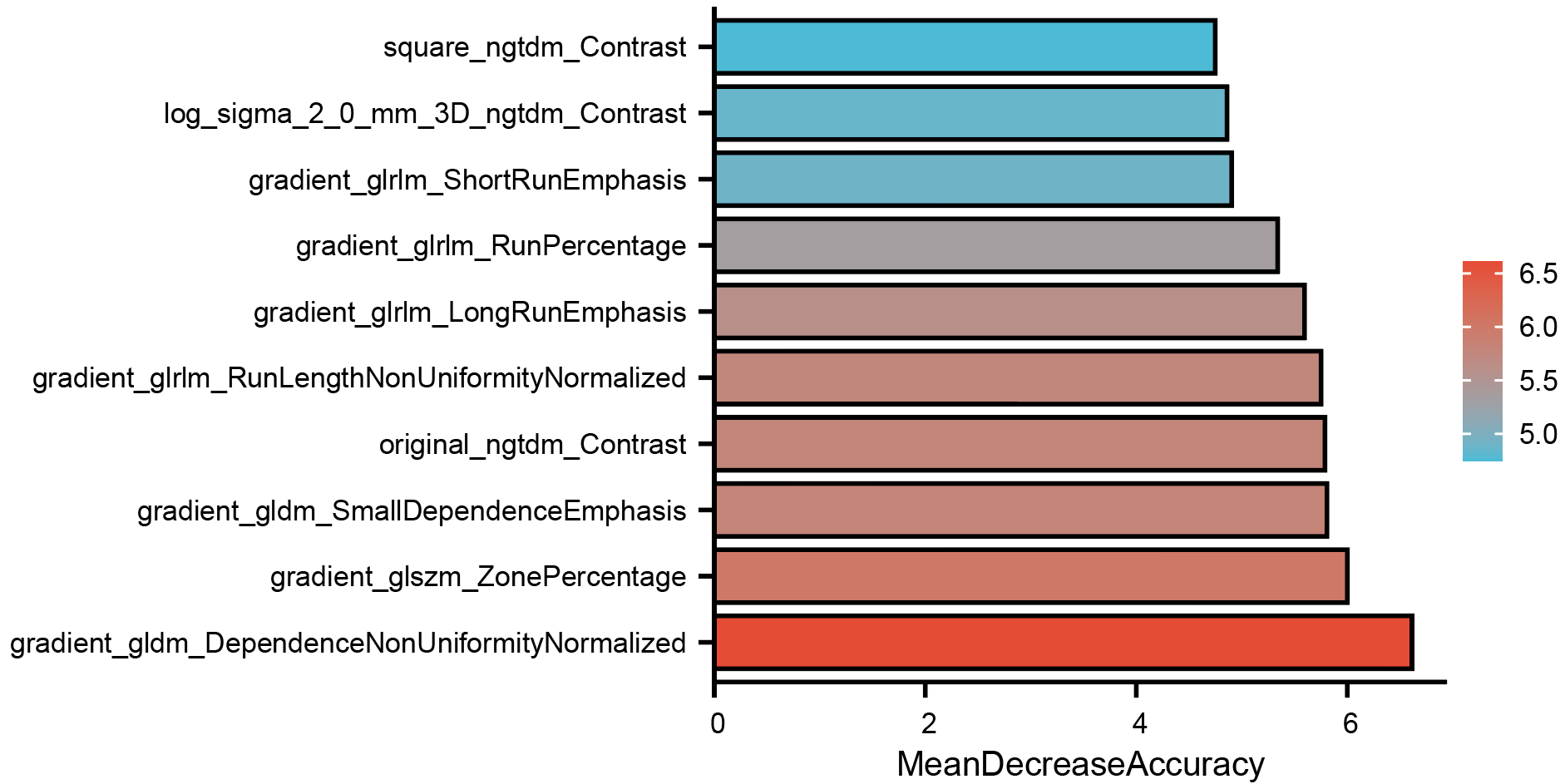


**Figure S5.** Random forest feature importance

## Figure S6. Re-clustering in combined cohort

##
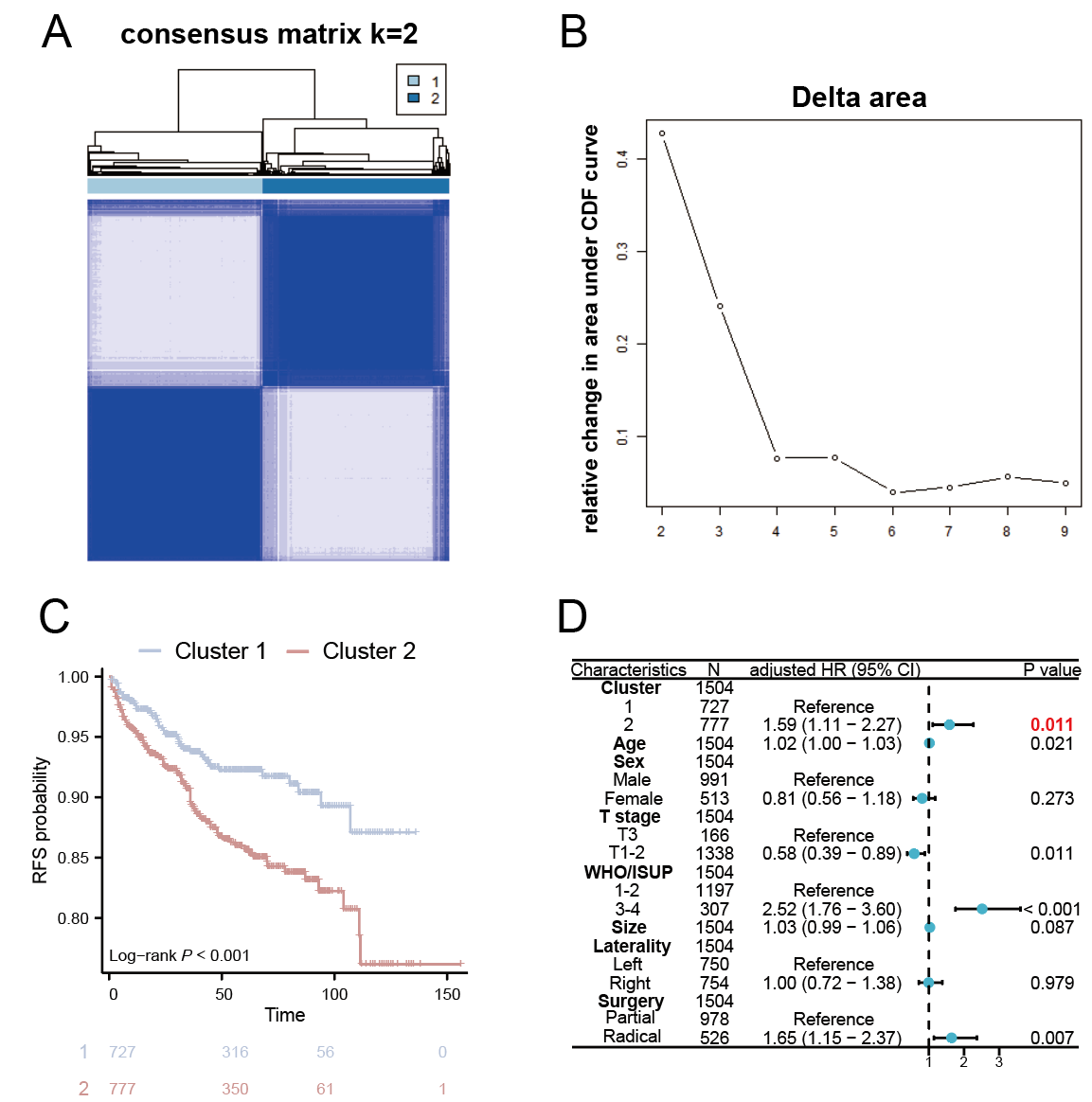


**Figure S6.** (A) Consensus matrix of 1504 ccRCC patients in discovery cohort when k = 2 using K-means clustering (Euclidean distance and 1500 iterations). (B) Cumulative distribution function. (C) The Kaplan-Meier survival curves compare RFS between new Cluster 1 (n=727) and new Cluster 2 (n=777) in the combined cohort. (D) Forest plot summarized independent predictors of RFS after adjusting for clinical information and Radiomic Cluster emerged a strong prognostic factor.

## Figure S7. The top 20 mutated genes were visualized by waterfall plot


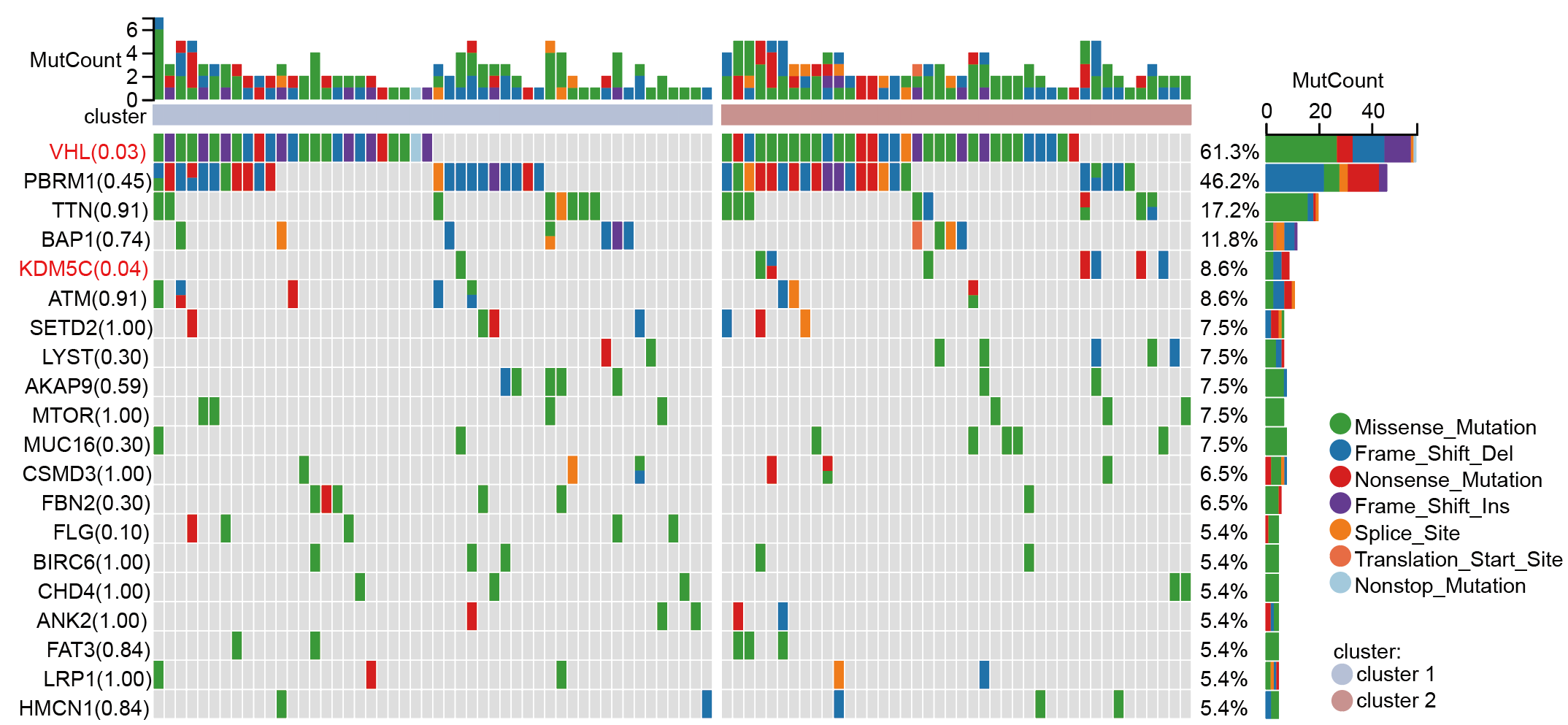


**Figure S7**. The top 20 mutated genes were visualized by waterfall plot between Cluster 1 and Cluster 2.

## Figure S8. GSEA showed downregulation of TCR Signaling pathways in Cluster 2


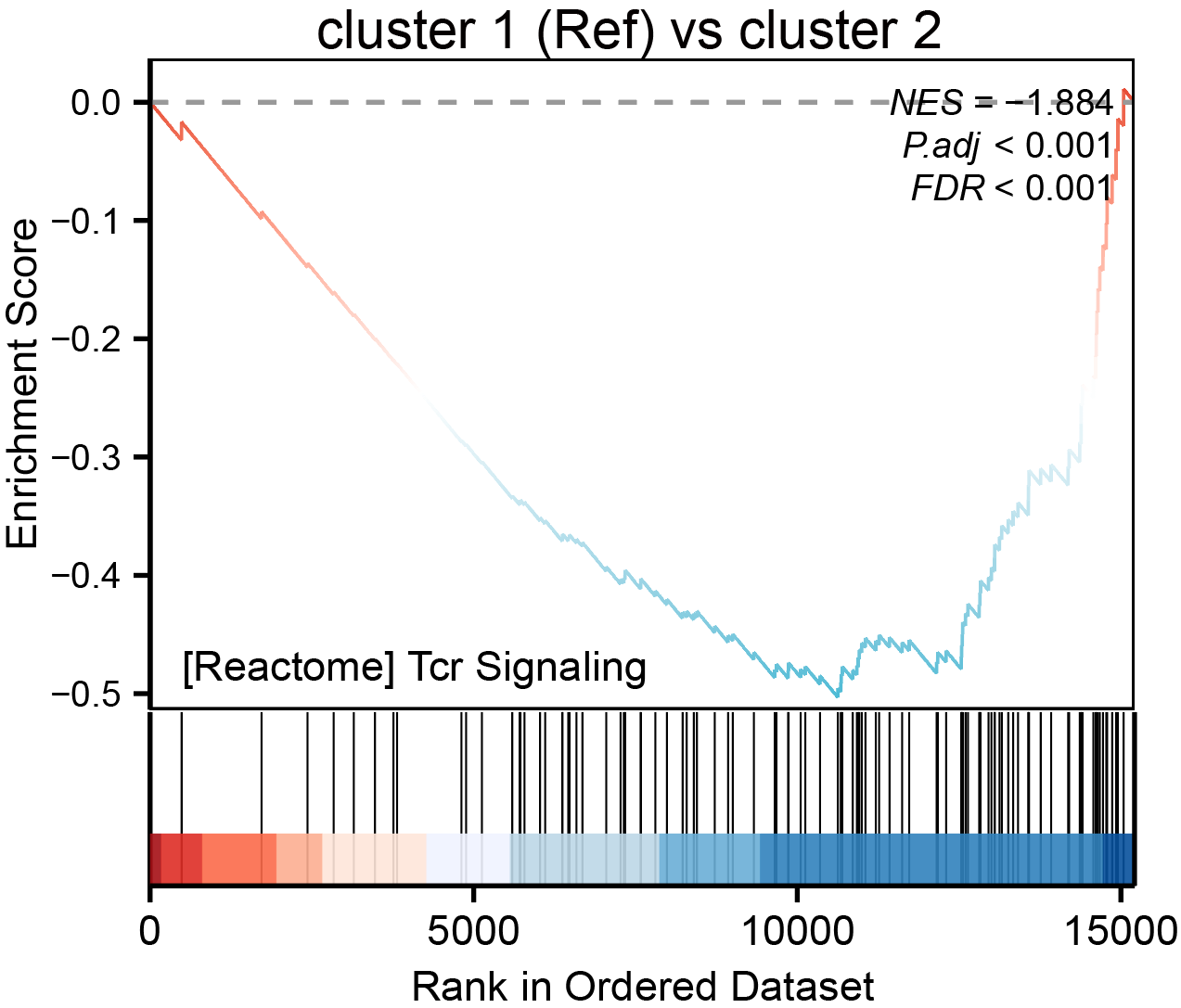


**Figure S8.** GSEA showed downregulation of TCR Signaling pathways in Cluster 2.

## Figure S9. GSEA of Hallmark gene sets


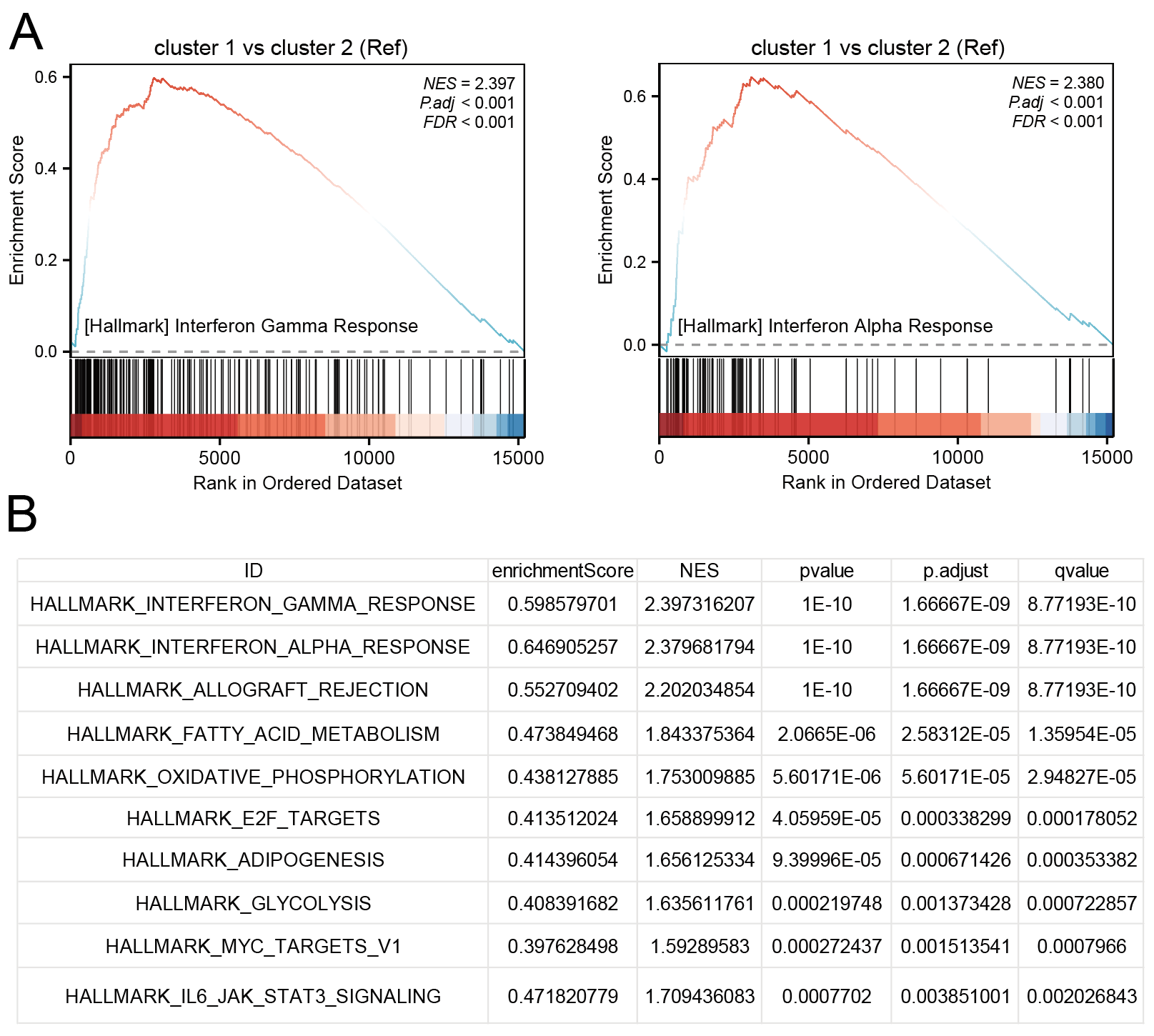


**Figure S9.** GSEA of Hallmark gene sets comparing Cluster 1 and Cluster 2 (Ref). (A) Enrichment plots of the Interferon Gamma Response and Interferon Alpha Response hallmark gene sets from GSEA. (B) Summary of top 10 results obtained through the GSEA analysis.

## Figure S10. The difference of MSI MANTIS Score between Cluster 1 and Cluster 2


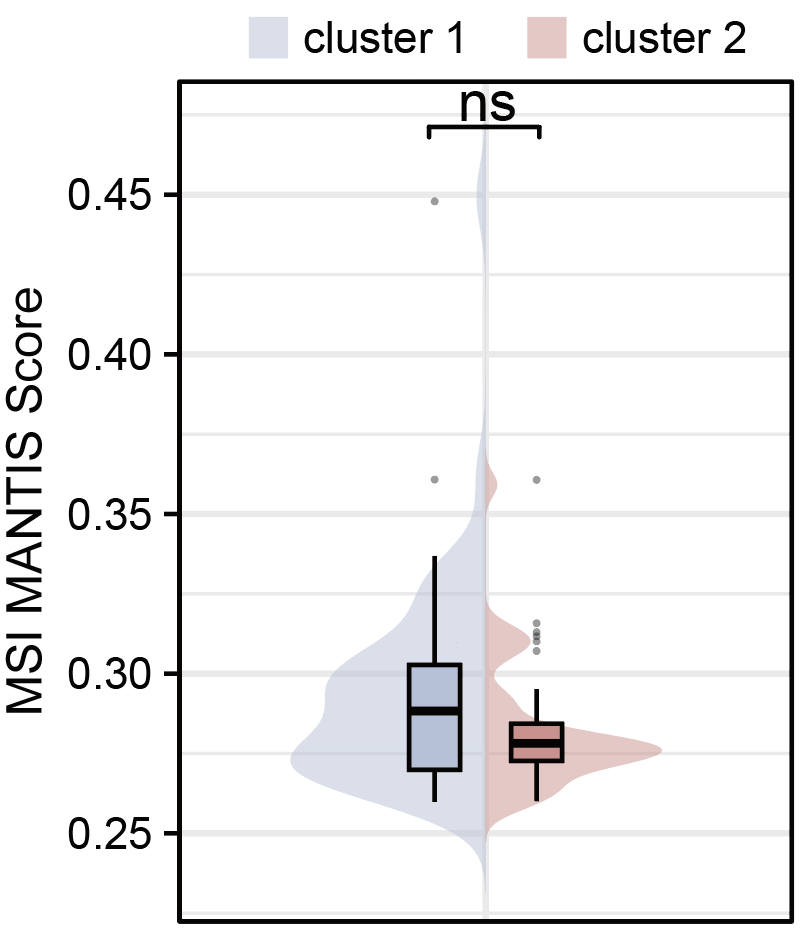


**Figure S10.** The difference of MSI MANTIS Score between Cluster 1 and Cluster 2.

## Supplementary Tables

## **Table S1. CT scanners and parameters of the three cohorts**

|  | JPH cohort | WHUH cohort | SDH cohort |
| --- | --- | --- | --- |
| CT scanners | Siemens, Toshiba, and GE | Siemens, Philips, Toshiba, and GE | GE |
| Slice thickness | 0.625-5 mm | 0.625-5 mm | 1.25 mm |
| Slice interval | 1-1.5 mm | 1-1.5 mm | 1-1.5 mm |
| Detector rows | 128, 256-section | 64, 128, 256-section | 64, 128-section |
| Tube current | Automatic  tube-current | Automatic  tube-current | 148-252 mA |
| Tube voltage | 120 kV | 70-140 kV | 120 kV |
| Data matrix | 512 * 512 | 512 * 512 | 512 * 512 |
| Contrast agent concentration | 350mgI/mL | 300~350mgI/ml | 300~350mgI/ml |
| Contrast agent dosage | 1.5ml/kg | 1.5ml/kg | 1.5ml/kg |
| Contrast agent infused rate | 2.5 ml/s | 2~3ml/s | 2~3ml/s |
| Arterial phase scan | 10s after aorta  reached trigger  100Hu | 10s after aorta  reached trigger  100Hu | 10s after aorta  reached trigger  100Hu |

Note. JPH：Jiangsu Provincial People’s Hospital. WHUH: Union Hospital, Tongji Medical College, Huazhong University of Science and Technology.

SDH: First Affiliated Hospital of Shandong First Medical University

## **Table S2. Baseline information of WHUH T-I cohort**

| **Characteristics** | WHUH T-I cohort |
| --- | --- |
| **Total (n)** | 39 |
| **Age, median (IQR)** | 64 (58, 68) |
| **Sex** |  |
| Male | 32 |
| Female | 7 |
| **cT stage** |  |
| 1 | 10 |
| 2 | 13 |
| 3 | 11 |
| 4 | 5 |
| **Laterality** |  |
| Left | 20 |
| Right | 19 |

Note. IQR: Interquartile range.
